# Idiopathic pulmonary fibrosis: Addressing the current and future therapeutic advances along with the role of Sotatercept in the management of pulmonary hypertension

**DOI:** 10.1002/iid3.1079

**Published:** 2023-11-09

**Authors:** Dalia D. Hadi, Mohammed Dheyaa Marsool Marsool, Ali Dheyaa Marsool Marsool, Neel Vora, Sajjad G. Al‐Badri, Nabeel H. K. Al‐Fatlawi, Ameer F. Abbas Al Wssawi, Abdullah M. T. Al‐Ibraheem, Khadija A. Hamza, Priyadarshi Prajjwal, Mohammed A. Mateen, Omniat Amir

**Affiliations:** ^1^ Department of Internal Medicine Al‐Kindy College of Medicine, University of Baghdad Baghdad Iraq; ^2^ Department Internal Medicine B.J. Medical College Ahmedabad India; ^3^ Department of Internal Medicine University of Baghdad, College of Medicine Baghdad Iraq; ^4^ Department of Internal Medicine University of Al‐Qadisiyah College of Medicine Diwaniya Iraq; ^5^ Department of Internal Medicine Bharati Vidyapeeth University Medical College Pune India; ^6^ Department of Internal Medicine Shadan Institute of Medical Sciences Teaching Hospital and Research Center Hyderabad India; ^7^ Department of Internal Medicine Al Manhal Academy Khartoum Sudan

**Keywords:** idiopathic pulmonary fibrosis, management, pulmonary hypertension, Sotatercept

## Abstract

**Background:**

Idiopathic pulmonary fibrosis (IPF) is a progressive and debilitating lung disease characterized by irreversible scarring of the lungs. The cause of IPF is unknown, but it is thought to involve a combination of genetic and environmental factors. There is no cure for IPF, and treatment is focused on slowing disease progression and relieving symptoms.

**Aims:**

We aimed in this review to investigate and provide the latest insights into IPF management modalities, including the potential of Saracatinibas a substitute for current IPF drugs. We also investigated the therapeutic potential of Sotatercept in addressing pulmonary hypertension associated with IPF.

**Materials and Methods:**

We conducted a comprehensive literature review of relevant studies on IPF management. We searched electronic databases, including PubMed, Scopus, Embase, and Web of science.

**Results:**

The two Food and Drug Administration‐approved drugs for IPF, Pirfenidone, and Nintedanib, have been pivotal in slowing disease progression, yet experimental evidence suggests that Saracatinib surpasses their efficacy. Preclinical trials investigating the potential of Saracatinib, a tyrosine kinase inhibitor, have shown to be more effective than current IPF drugs in slowing disease progression in preclinical studies. Also, Sotatercept,a fusion protein, has been shown to reduce pulmonary vascular resistance and improve exercise tolerance in patients with PH associated with IPF in clinical trials.

**Conclusions:**

The advancements discussed in this review hold the promise of improving the quality of life for IPF patients and enhancing our understanding of this condition. There remains a need for further research to confirm the efficacy and safety of new IPF treatments and to develop more effective strategies for managing exacerbations.

## Graphical abstract

This review examines idiopathic pulmonary fibrosis (IPF) treatments, including Pirfenidone and Nintedanib, which reduce fibrosis and preserve lung function. Saracatinib demonstrates greater efficacy, while emerging therapies are also explored. Further research is required to address gaps in IPF management, such as exacerbations and associated pulmonary hypertension. Promising results are observed in clinical trials investigating Sotatercept as a potential treatment for pH in IPF patients

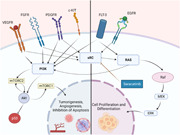

## 1 INTRODUCTION

A recent understanding of idiopathic pulmonary fibrosis (IPF) states that IPF is a progressive disease of the lung interstitium mainly represented by fibrous remodeling of the alveoli as well as a gradual loss of irreversible pulmonary function. The process is thought to be an accumulation of extracellular matrix (ECM) over the long term. Permanent inhibition of oxygen transfer occurs due to this accumulation, which causes shortness of breath.^1^, ^2^, ^3^, ^4^, ^5^

The precise causes of histopathological changes in IPF remain unknown. IPF is associated with various risk factors like smoking, infections, pollutants, genetics, and drugs.^6^, ^7^ However, these factors do not fully explain the extensive remodeling and progression of IPF, or its increased incidence with age. IPF may start in susceptible individuals due to nonspecific damage to the lung's epithelial barrier and parenchyma caused by these risk factors. There's a debate about whether inflammation precedes fibrosis. Animal studies show that inflammation often comes before fibrosis, and alveolitis in early pulmonary fibrosis involves various inflammatory cells.^8^, ^9^ Even asymptomatic relatives of IPF patients can display signs of alveolitis.^10^ Alveolar macrophages, which release proinflammatory and profibrotic cytokines, are thought to play a role in IPF.^11^, ^12^ However, this view is disputed because inflammation is generally minimal in IPF patients, and fibrosis can occur without inflammation.^13^, ^14^, ^15^ Anti‐inflammatory treatments like systemic glucocorticoids have not been effective and sometimes worsen the condition.^16^ In our study, we explore a crucial dimension of pulmonary fibrosis, addressing a pressing issue in the field of respiratory medicine. IPF presents a complex challenge for healthcare professionals, with limited treatment options available. Our research aims to bridge this gap by focusing on the development and evaluation of novel therapeutic agents. Specifically, we investigate interventions designed to reduce pulmonary fibrosis by targeting fibroblast activity and intervening with cell signaling pathways. Furthermore, our study contributes to the ongoing discourse in the field, inspiring further research and innovation in the management of this challenging condition. Furthermore, we attempted to address the effect and the potential of Sotatercept in treating PAH associated with IPF.

### 1.1 Immunological interactions, cytokines, and genetics in IPF

In the context of immunity, interactions between growth factors, cytokines, and lung‐resident cells are crucial in the evolution of fibrotic responses in IPF.^17^, ^18^, ^19^ Lung‐resident cells, including epithelial cells, fibroblasts, and endothelial cells, produce cytokines and growth factors that stimulate fibroblast proliferation and matrix synthesis. Following lung epithelial injury, fibrosis progresses due to imbalances in proinflammatory and anti‐inflammatory cytokines, fibrogenic and antifibrogenic polypeptides, oxidants‐antioxidants, and angiogenic and angiostatic molecules.^20^ In contrast to bronchoalveolar lavage (BAL) samples from healthy individuals, BAL specimens from individuals with IPF exhibit heightened levels of transforming growth factor‐β (TGF‐β), including TGF‐β1.^21^, ^22^ TGF‐β1 also possesses the capability to induce the production of several growth factors and cytokines involved in fibrosis, such as connective tissue growth factor (CTGF), FGF‐2, PDGF, and insulin‐like growth factor (IGF).^23^ Experimental evidence supports the role of TGF‐β in IPF, as reducing its expression, signaling, and activity has been shown to decrease fibrosis.

Another cytokine that has been involved in IPF pathogenesis is tumor necrosis factor‐α (TNF‐α). TNF‐α expression is elevated in IPF, but its specific role is not clear yet. TNF‐α can increase TGF‐β1 production, stimulate fibroblast proliferation, and induce collagen synthesis^24^, ^25^ In mice with fibrosis, TNF‐α reduces fibrotic burden and improves lung function. Conversely, IFN‐γ, known to inhibit fibroblast proliferation and connective tissue synthesis, appears deficient in IPF lungs, although treatment with INF γ‐1b does not reduce IPF mortality.^26^, ^27^, ^28^, ^29^, ^30^

In IPF, there is an excessive expression of fibrosis‐inducing cytokines. The potential role of molecules such as monocyte chemoattractant protein 1 (MCP‐1) in attracting IPF cells is gaining recognition. Among these cytokines, TGF‐beta, CTGF, IL‐4, IL‐13, FGF‐2, IGF‐1, PDGF, and GM‐CSF promote fibrosis, while IFN‐gamma, IL‐1, IL‐10, IL‐12, and IL‐17 have anti‐fibrotic effects. The progression of fibrosis seems to involve the activator protein (AP)‐1 transcription factor and the fos‐related protein Fra‐2. Gene expression profiling has revealed additional proteins associated with IPF pathogenesis and disease activity.^31^, ^32^, ^33^, ^34^, ^35^, ^36^ For instance, genes like MMP‐7, MMP‐1, surfactant protein A1, cyclin A2 (CCNA2), and alpha‐defensins are overexpressed in lung tissue and blood of IPF patients.^33^, ^34^, ^35^ Analyzing these profiles alongside genes linked to lung development may offer insights into the disease's mechanisms.^31^, ^32^ Additionally, increased MMP‐7 and MMP‐1 levels in peripheral blood may correlate with disease activity.^36^

### 1.2 Clinical features, prognosis, and current management modalities

The major characteristics of the disease are cough and dyspnea which both have a bad impact on the patients in terms of quality of life^37^, ^38^ in addition to its effect on life expectancy, with a 3‐year median survival when left untreated.^39^ What defines IPF is the histopathological and radiological features of lung tissue described as extensive deposition of the ECM that leads to changes in lung architecture, increased alveolar wall thickness, and dilated bronchi.^40^ Cases of suspected IPF can be assessed by chest high‐resolution CT scan.^41^, ^42^ IPF has been found to be the most prevalent type of interstitial lung disease constituting about 17%–37% of all interstitial lung diseases^43^ which makes it mandatory to look for the best treatments possible for the disease. For many years, the management of patients with IPF was only symptomatic, and for decades now, the only way to cure IPF is lung transplantation, but in the last decade, multiple drugs have been undergoing clinical trials aiming at better outcomes in IPF patients. Until recently, only two of them have been validated and proven effective in slowing the progression of IPF, Nintedanib, and pirfenidone, while other management modalities are still being tested for their effectiveness.

### 1.3 Methodology

#### 1.3.1 Literature search strategy

To compile a comprehensive narrative review on IPF, we conducted an extensive literature search. The search was performed across various electronic databases, including PubMed, Scopus, Embase, and Web of Science. We employed a combination of keywords and Medical Subject Headings (MeSH) terms such as “Idiopathic Pulmonary Fibrosis,” “pulmonary fibrosis,” “Management,” “Sotatercept,” “pulmonary hypertension,” “IPF pathogenesis,” “IPF treatment,” and related terms.

#### 1.3.2 Inclusion and exclusion criteria

Articles included in this review were primarily selected based on their relevance to the topic of IPF, its pathogenesis, current treatment options, and the role of Sotatercept in managing pulmonary hypertension (PH) in IPF patients. We focused on articles published in peer‐reviewed journals, clinical trials, systematic reviews, and meta‐analyses. Studies and reports not written in English were excluded.

## 2 RESULTS

### 2.1 Antifibrotic agents

#### 2.1.1 Pirfenidone

A drug with antifibrotic properties used now for IPF known as Pirfenidone or PFD, is taken orally.^44^ This drug is a synthetic molecule that is small with a characteristic of rapid absorption in the gastrointestinal tract and an estimated 3‐h half‐life.^45^ Its metabolism is mainly by cytochrome P450, which occurs in the liver and most of it is excreted in the form of 5‐carboxy‐pirfenidone in urine (80%) or in feces (20%). PFD was found to have two effects on the body, antifibrotic and anti‐inflammatory effects.^46^ PFD has an inhibitory effect on the proliferation of fibroblasts as well as the synthesis of collagen which is done through interfering with the signaling of TGF‐β, and other growth factors, like basic fibroblast growth factor and PDGF.^47^, ^48^ It has been shown that PFD is a strong inhibitor of fibronectin and the production of α‐smooth muscle actin (α‐SMA) which is known to have a role in fibro‐myofibroblast transition, when set with TGF‐β. PFD has the ability to inhibit TGF‐β mediated fibrotic changes in human fetal lung fibroblasts.^49^, ^50^

PFD has also shown its anti‐inflammatory effect following allergen‐induced presensitization, decreasing airway responsiveness, inflammatory cytokines, and cells in the bronchoalveolar fluid.^51^, ^52^ Another study revealed that PFD might have the potential to reduce the generation of proinflammatory cytokines by stopping the action of p38 MAP Kinase in B lymphocytes, introducing a new potential in PFD for lung fibrosis, as migration and activation of fibroblasts can occur due to the inflammatory process started by B‐cell‐derived cytokines.^53^ While the ASCEND study examined PFD therapy for 52 weeks, the CAPACITY studies assessed its effectiveness and safety for a minimum of 72 weeks. Adverse effects of PFD were found to be mostly photosensitivity, nausea, skin rash, gastrointestinal upset, and anorexia. Serious adverse effects included irregular liver function, facial palsy, dizziness, and hepatocellular tumor.^54^

The approved dose of pirfenidone that is recommended in Asia is 1800 mg per day, while 2403 mg per day is acceptable in Europe and the United States.^55^ A study was conducted to evaluate the efficacy of lowered doses of PFD in which the results showed that patients managed with a lower dose of PFD had the same clinical outcomes compared to other patients taking the standard‐doses of PFD and so, minimizing the dose could be helpful to maintain the therapeutic efficacy while managing the adverse effects at the same time.^55^

#### 2.1.2 Nintedanib

Nintedanib, or NDB, is taken orally and has antifibrotic activity by inhibiting tyrosine kinase receptors like FGFR, PDGFR, and VEGF receptors, thus inhibiting the signaling pathways of FGF, PDGF, and VEGF.^3^, ^56^ NDB also has anti‐inflammatory activities, although it still needs full comprehension. Studies showed that it acts by inhibiting mediators, including IL‐2, 4, 5, 10, 12p70, 13, and IF‐γ by mononuclear cells in the peripheral blood or T cells in the human body.^57^ NDB was first developed for antitumor purposes and was of the first drugs labeled as FDA‐approved for IPF in the Europe and United States, along with PFD, after the success of the INPULSIS and INSTAGE trials (NDB showed effectiveness in decreasing the decline in forced vital capacity).^58^, ^59^, ^60^, ^61^ The FVC in UIP patients stabilized as a result of this observational study's findings, and the safety profile sounded better than that of the SENSCIS trials. Our data also suggested good safety, even in patients taking anticoagulants.^62^ Treatment with nintedanib was tolerable in elderly IPF patients and suitable in the therapeutic adverse event management. Additionally, nintedanib could be utilized by elderly IPF patients as chronic treatment. When starting treatment, careful patient management might be required with nintedanib in patients with advanced IPF.^63^

A 150 mg of NDB can provide a therapeutic effect close to the maximum NDB effect, regardless of disease condition or demographic baselines, for most patients with IPF.^64^ The adverse effects of the drug were mainly diarrhea, nausea, nasopharyngitis, cough, and vomiting.^65^ As the elimination of the drug is primarily (>90%) biliary/fecal, with a negligible role of renal excretion,^66^ a lowered dose of 100 mg BID for patients with mild hepatic problems or patients experiencing adverse effects is advised.^65^

## 3 POTENTIAL AND FUTURISTIC DRUGS IN THE MANAGEMENT OF IDIOPATHIC PULMONARY FIBROSIS

### 3.1 Saracatinib

**Figure 1:**
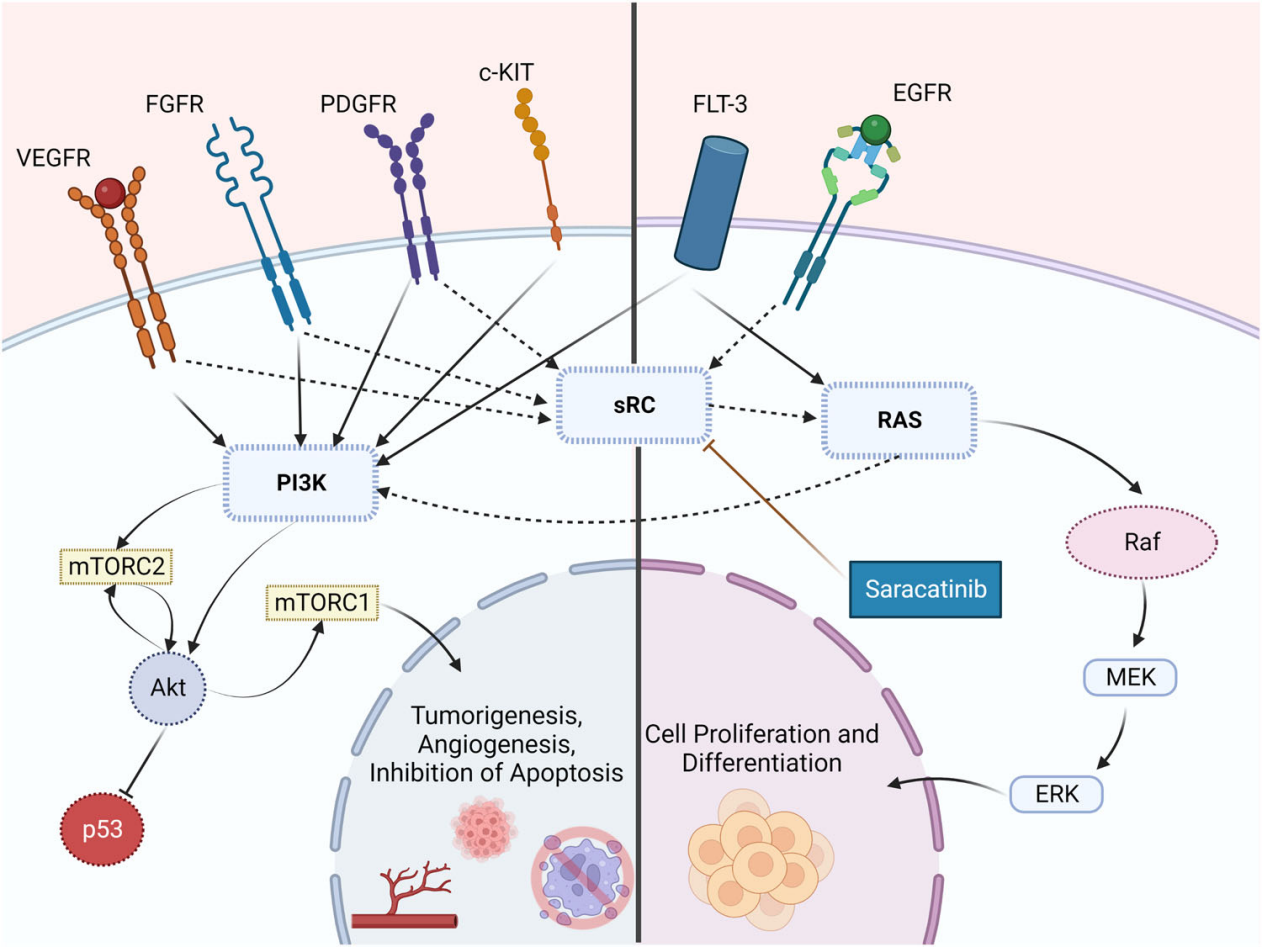
Saracatanib mechanism of action. Saracatinib acts by the inhibition of the SRC protein, resulting in a reduction of signaling pathways. This subsequently diminishes RAS activation, ultimately inhibiting the proliferative activity and the differentiation in fibroblasts. c‐KIT, tyrosine‐protein kinase kit; EGFR; epidermal growth factor receptor; ERK, extracellular signal‐regulated kinase; FGFR, fibroblast growth factor receptor; FLT‐3; FMS‐like tyrosine kinase 3; MEK, mitogen‐activated protein kinase; PDGFR, platelet‐derived growth factor receptor; sRC, sarcoma tyrosine kinase; VEGFR, vascular endothelial growth factor receptor.

Saracatinib is a specific, strong inhibitor of the Src kinases that was first developed for antitumor purposes.^67^ Saracatinib has demonstrated that it inhibits the induced increase in the activity of Src kinase in fibroblasts by TGF‐β.^67^ Saracatinib's efficacy in treating IPF patients is currently under investigation by a Phase 1b/2a clinical trial (STOP‐IPF), as the safety profile is now already established. Saracatinib inhibits sRC causing a decrease in the signaling pathway of VEGF, FGF, PDGF, and EGF further decreasing RAS activation, finally leading to the inhibition of the proliferation of fibroblasts as well as its differentiation (Figure 1).

Saracatinib has a larger inhibitory effect than the other two antifibrotic medications on the expression of various profibrotic genes induced by TGF‐β, like ACTA2, SERPIN1, and COL1A1, according to an in vitro model research that was conducted with an aim of comparing the efficacy of Saracatinib with the other approved antifibrotic medications. In addition, Saracatinib inhibits TGF‐Β leading to a change in many signaling pathways, including the JAK‐STAT3, IL6, and IFN‐γ. It also inhibits the α‐SMA and filamentous actin (F‐actin). All of these inhibitory effects prevent fibroblast transformation to myofibroblast as well as decrease pulmonary collagen deposition.^67^

In a precision cuts lung slices (PCLS) conducted for complementary purposes, saracatinib had a much better effect than NDB and PFD by studying an ex vivo model showing reduction in pulmonary fibrosis and this was confirmed in in vivo mouse models.^31^ Therefore, this study provided an absolute indication that saracatinib is equal to or could be even better than the currently used antifibrotic drugs, PFD and NDB as an inhibitor of pulmonary fibrosis in experimental models,^31^ showing a potential that it could replace both drugs in the future (Tables 1 and 2).

**Table 1:** Comparison between Saracatinib, Nintedanib, and Pirfenidone.

Comparison aspect	Saracatinib	Ninetidanib	Pirfenidone
Mechanism of action	Src kinase inhibitor	Tyrosine kinase inhibitors	TGF‐β and fibronectin inhibitor as well as p38 MAPK in B lymphocytes
TGF‐β expression of profibrotic genes	Larger inhibitory effect including ACTA2, COL1A1, and SERPIN1	Inhibits TGF‐β expression of profibrotic genes	Inhibits TGF‐β expression of profibrotic genes
TGF‐β‐induced Smad3 phosphorylation	Inhibitory effect	No effect was found	No effect was found
Experimental pulmonary fibrosis reduction	Better effect	Less effect	Less effect
Precision cuts lung slices results	Better effect in reducing pulmonary fibrosis	Less effect	Less effect

Abbreviations: ACTA2, alpha‐smooth muscle actin; COL1A1, collagen type I alpha 1 chain; MAPK, mitogen‐activated protein kinase; TGF‐β, transforming growth factor‐β.

**Table 2:** Summary of the newer modalities in the treatment of IPF.

Name	Mechanism of action	Clinical trial	Effects	Side effects
Pirfenidone	TGF‐β and fibronectin inhibitor	CAPACITY/ASCEND	Antifibrinolytic and anti‐inflammatory, reduction in all‐cause mortality	Photosensitivity, nausea, skin rash, gastrointestinal upset, and anorexia. Serious adverse effects: abnormal liver function, dizziness, facial paralysis, hepatocellular tumor
Nintedanib	Tyrosine kinase inhibitors	INPULSIS/INSTAGE	Reducing FVC decline and all‐cause mortality	Diarrhea, nausea, nasopharyngitis, cough, and vomiting
Saracatinib	Src kinase inhibitor	STOP‐IPF	Better effect in reducing FVC decline than PFD and NDB	Gastrointestinal discomfort
Pamrevlumab	CTGF inhibitor	ZEPHYRUS I, II	Decrease in FVC decline, improvement in progression‐free survival.	Headache, cough, breathlessness, URTI, bronchitis.
Pentraxin‐2	Recombinant human pentraxin‐2, has an activity on monocyte differentiation	STARSCAPE‐OLE	Owing of futility, stopped	Cough, nasopharyingitis, fatigue
BI 1015550	phosphodiesterase 4B (PDE4B) inhibitor	PRAISE	Reduction in FVC decline with/without antifibrotic therapy	Mild diarrhea.
Ziritaxestat	selective autotaxin inhibitor	ISABELA 1, 2	Annual rate in FVC decline, but stopped due to concerning safety	Lower respiratory tract infections
PBI‐4050	interacting with GPR40 and GPR84 (activation and suppression respectively)	NCT02538536	Reduced histological lesions presented as change in lung architecture, thickness of the alveolar wall and fibrosis	Mild diarrhea, headache, and nausea
Bexotegrast	inhibitor of integrins v6 and v1	INTEGRIS‐IPF	Excellent safety profile Less progress was supported by exploratory analysis	Mild diarrhea
BMS‐986020/BMS‐986278	LPA1 antagonists	NCT04308681	Change in percent predicted (ppFVC)	Hepatobiliary toxicity in BMS‐986020
TD 139	Gal‐3 inhibitor	NCT02257177	Slower decline in FVC.	Taste disturbance and cough
Dasatinib (D) and Quercetin (Q)	Senescent cell antiapoptotic pathways (SCAPs) inhibitors	NCT02874989	Improved lung function and exercise capacity	Nausea, weakness, headache, sleep disturbance, and sepsis in patients with chronic cholelithiasis

Abbreviations: CTGF inhibitor, connective tissue growth factor inhibitor; GPR40, G‐protein‐coupled receptor 40; GPR84, G‐protein‐coupled receptor 84; LPA1 antagonists, lysophosphatidic Acid 1 Antagonists; TGF‐β, transforming growth factor‐β.

### 3.2 Pamrevlumab

A recombinant antibody called pamrevlumab has the capability to recognize the CTGF, binds to it, and therefore stops it from cytokines binding, thus avoiding following inflammatory signaling.^68^ The CTGF is released by multiple types of cells like fibroblasts, myofibroblasts, and endothelial cells and it's a glycoprotein. It is thought CTGF has interactions with different regulators, like TGF‐β, VEGF, and integrin receptors. This way, CTGF regulates the response of cells to the environment, such as secretions, sorting, production of ECM, motility of cells, and adhesion. These biological processes have been linked to cancer formation and abnormal tissue healing, including fibrosis.^69^ PRAISE trial (a phase 2 trial) showed in 2019 that pamrevlumab taken intravenously decreases the decline in FVC successfully by 70% in approximation in patients with IPF in comparison with those receiving placebo.^33^ Interestingly, the benefits from the treatment were seen, unlike any other trials, in spite of whether the change in FVC was expressed as an alteration in percentage predicted values, volume change, or in a categorical analysis of free‐of‐progress survival as the majority component.^70^ The impact of treatment was noticeable with equal treatment effects in radiological and symptomatic parameters, which was not found in other studies, yet the results should be treated cautiously until an appropriately powered phase 3 investigation is done.^69^ This study's findings are perhaps on the top of phase 2 trials results, and whether pamrevlumab's therapeutic advantages might be enhanced with the administration of antifibrotic medications is now under studies.^70^, ^71^

### 3.3 Pentraxin‐2

The naturally occurring protein called pentraxin‐2 has a recombinant form, named recombinant human pentraxin‐2 (rhPTX‐2; aka PRM‐151). This drug is being investigated for its potential to be a probable option in IPF treatment. A phase II trial, which was a double‐blind randomized, placebo‐controlled PRM‐151‐202 portion research that tested rhPTX‐2 in patients with IPF (NCT02550873), has shown results that are now published.^72^ When compared to placebo, rhPTX‐2 considerably slowed the decrease in FVC and stabilized 6‐min walk distance (6MWD) after receiving the drug, according to the placebo‐controlled study of PRM‐151‐202.^36^ For those patients receiving either PFD or NDB with it, as well as individuals receiving rhPTX‐2 monotherapy, efficacy patterns of rhPTX‐2 were seen.^72^ This Phase II study's observation of rhPTX‐2's impact on 6MWD was novel because this is the first time a clinical trial for IPF uses a 6MWD to demonstrate the stability of functional status of patients.^72^, ^73^

In the group taking the medication, a decrease in the drop in the mean percent predicted FVC and 6MWD in meters was shown, and this was maintained for up to 52 weeks.^72^, ^74^ When patients started taking Pentraxin‐2 during the extension phase of the study, the percent of their FVC decline was enhanced from 8.7% a year to 0.9% a year and their 6MWD leveled up from 54.9 meters a year to 3.5 m a year. However, the long‐term consequences of IPF were still consistent with the results, occurring in about 28% of patients.^74^ These results will be explored more in a 52‐week Phase III research of rhPTX‐2 (STARSCAPE), a long‐term OLDE study will also follow the phase III trial, to evaluate the clinical importance. If the results of the Phase III study are consistent with the results of the Phase II trial, rhPTX‐2 could be very promising as an adjunctive option to current antifibrotic drugs to delay IPF progression as well as an efficient monotherapy option in patients who are unable to tolerate the available choices.^74^

### 3.4 BI 1015550

One of the drugs that are now being investigated is the inhibitor of phosphodiesterase 4 group, BI 101550. Phosphodiesterase 4, or PDE4, is described by proteins that play crucial roles in the cells of a human being. Drugs that suppress PDE4 activity have been demonstrated in prior research to reduce inflammation and scarring.^75^ Inhibition of PDE4 leads to the inhibition of fibroblast action, further preventing the transformation of fibroblast to myofibroblast. The PDE4 group has many functions, inhibiting all of its functions could be problematic as it may cause side effects. This drug primarily works on PDE4B, and it is anticipated by researchers that this will decrease the likelihood of side effects. A phase II double‐blinded, randomized study was done and the drug BI 1015550 was examined for having the potential to become an option in managing IPF. The study compared the placebo with BI 1015550.^75^ In total, 147 IPF patients from 22 different countries participated in the trial. The findings demonstrated that BI 1015550 protected IPF patients' lung function from deteriorating. With BI 1015550 or placebo, no difference was seen in patients with medical conditions the study physician classified as severe. However, diarrhea affected more persons who received BI 1015550 treatment. Thirteen patients receiving BI 1015550 had to stop their treatment because of health problems; none of the patients receiving a placebo had to stop their treatment because of health problems.^75^

### 3.5 Ziritaxestat

Lysophosphatidic acid (LPA) is hypothesized to at least in part mediate the abnormal wound healing responses that lead to fibrosis that is seen with IPF. IPF patients have higher levels of LPA and the enzyme responsible for its formation, autotaxin (ATX), demonstrating their involvement in the etiology of the disease and suggesting possible targets for novel therapeutics.^76^

Phase IIa research was done involving 23 IPF patients. Ziritaxestat, which is a small‐sized molecule of selective autotaxin inhibitor,^77^, ^78^ demonstrated promising outcomes.^79^ When compared to placebo at Week 12, those on ziritaxestat showed a reduced change in FVC. Ziritaxestat here was well tolerated. Ziritaxestat also decreased plasma LPA concentration, showing a maximal decline from the baseline of almost 90%, indicating target reach.^79^ Phase 3 of two randomized clinical studies, ISABELA 1 and 2, with identical designs, had been done to further assess the effectiveness and safety of ziritaxestat in IPF. In this trial, in patients getting treatment with PFD or NDB or in those not receiving the PFD/NDB treatment, ziritaxestat did not prove to have a better clinical outcome compared to placebo.^80^ That's part of the reason why the ISABELAs failed, which needs to be looked into more. This could be looked into by continuing research on other autotaxin inhibitors, such as BBT‐87723, or LPA receptor antagonists, such as BMS‐98627824, which have different pharmacological properties from those in ziritaxestat. It should be noted that the LPA receptor antagonist (BMS‐986020) was halted because of the resultant hepatobiliary toxicity, but it was later determined that this was unrelated to LPA antagonism^81^, ^82^ in fact, no such safety concerns were detected in the ISABELAs.

### 3.6 PBI‐4050

PBI‐4050, which is an orally active low molecular weight chemical considered first‐in‐class, is being tested in trials for the treatment of disorders of fibrosis including IPF. It is the sodium salt of 3‐pentylbenzeneacetic acid. It is an artificial form of a medium‐chain fatty acid that binds to the G‐protein coupled receptors GPR40 and GPR84 with agonist and antagonist affinities, respectively. By regulating fibroblasts/myofibroblasts, macrophages, and epithelial cells, it can reduce or reverse fibrosis.^83^ By interacting with GPR40 and GPR84, PBI‐4050 stops the progression of fibrosis by regulating a number of antifibrotic pathways through this interaction connected to the emergence of IPF.^83^ The absence of the expression of alpha‐smooth muscle actin in fibroblasts and the concomitant increase of ECM deposition and fibrosis are a shred of evidence that the drug prevents the differentiation of fibroblasts into myofibroblasts. Monocyte chemoattractant protein‐1, IL‐8, and 6 which have the major role in inflammatory processes in addition to CTGF which has a major role in developing IPF all had been decreased by PBI‐4050.^83^ PBI‐4050 also dramatically reduces fibrosis in bleomycin‐induced lung fibrosis in a murine model as well as models in the heart, liver, lung, kidney, pancreas, and skin.^83^ The drug caused a 47% reduction in lung tissue disruption, fibrosis, as well as alveolar wall thickness.^83^ According to these findings, PBI‐4050 may have clinical benefits for fibrotic disorders like IPF. A phase II single‐arm open‐label research (NCT02538536) was carried out for 12 weeks at six sites across Canada in people with IPF.^84^ The main goal of this study was to assess PBI‐4050s safety and tolerability in this patient population. The findings of this trial demonstrated that there were no safety concerns following 12 weeks of management in patients with primarily mild to moderate IPF, whether used as monotherapy or combined with nintedanib or pirfenidone. PBI‐4050s PK profiles were identical when used alone and in conjunction with nintedanib, but they were altered when combined with pirfenidone, pointing to a potential interaction between drugs. FVC outcomes for PBI‐4050 by itself and when combined with nintedanib were promising.^84^

### 3.7 Bexotegrast

Or PLN 74809, an oral small molecule had been confirmed in vivo to have antifibrotic by inhibiting dual αvβ6/αvβ1 integrin, this results in the partial inhibition of the TGF‐β signaling pathway. By this mechanism of action, Bexotegrast can be used for the reduction of systemic side effects and toxicities produced by the full TGF‐β signaling pathway inhibition in the treatment of IPF, as well as inhibiting the expression of mRNA collagen.^85^ A phase IIa, open, four‐part, double‐blind, randomized, placebo‐controlled study known as INTEGRIS‐IPF is now investigating the safety, tolerability, and pharmacokinetics of PLN 74809. Good efficacy, safety, and tolerance were shown by PLN 74809. For all patients receiving PLN‐74809, the average decrease in FVC was 15.1 mL in contrast to 74.1 mL for individuals receiving the placebo. With larger doses of the medication, the FVC decline was improving, and in line with the encouraging outcomes seen thus far for larger doses, Pliant has disclosed a trial extension that assesses the effectiveness of 320 mg of PLN‐74809 administered daily for 6 months to persons with IPF. Early in 2023, preliminary trial results ought to be made public.^86^

### 3.8 BMS‐986020

In a phase II study, the first‐generation drug, BMS‐986020, which is an oral LPA1 antagonist, showed mechanism proof in patients with IPF.^87^ In general, BMS‐986020 decreased FVC decline during the course of 26 weeks when compared to placebo, with substantial changes occurring after 600 mg twice daily (BID) treatment. BMS‐986278 which is the second generation of LPA1 antagonist, is being developed to treat people with IPF. In contrast to BMS‐986020, in vitro research demonstrates that BMS‐986278 shows no inhibitory effect on the transporters of liver efflux, specifically the multidrug resistance 3 (MDR3) and the bile salt export protein (BSEP). Additionally, in vivo testing and phase 1 studies have not revealed any signs of direct hepatobiliary toxicity.^88^, ^89^ This phase II trial's aim is to assess BMS‐986278 in IPF patients or IPF‐ILD patients given that the antagonism of LPA1 was proven to be helpful in IPF patients.

### 3.9 TD 139

The expression of galectin (Gal)‐3, a key regulator of lung fibrosis, is raised in the lavage fluid and serum of the bronchi and alveoli of IPF patients, and this expression is further elevated during acute phases.^90^, ^91^ In mouse models of pulmonary fibrosis, TD139, a Gal‐3 inhibitor with a strong affinity for the carbohydrate recognition domain of Gal‐3, has demonstrated effectiveness.^90^, ^91^ The key to TD139's antifibrotic potential is the inhibition of recruitment and growth of Gal‐3‐secreting macrophages, which promote local myofibroblast activation.^92^, ^93^ Preclinical studies have demonstrated that TD139 is effective on all of the major IPF cell types, including fibroblast activation, Gal‐3/macrophage phenotype expression, a reduction in the activity of important profibrotic growth factors on myofibroblasts, and epithelial‐mesenchymal transition inhibition.^90^, ^91^, ^93^ A phase 1/2a, multicenter, randomized trial conducted in the United Kingdom assessed the effectiveness of TD 139. With the exception of TD139 being less preserved in the lungs of patients with IPF, the pharmacokinetic characteristics were basically similar between the healthy and IPF participants. Additionally, TD139 was found to have good tolerance by both healthy people and IPF patients, with the most common side effects being disturbance of taste (36.1%) and cough (11.1%). No significant changes on clinical terms were found in electrocardiographs, nor any hematological, biochemical markers, or clinical findings.^94^

### 3.10 Dasatinib (D) and quercetin (Q)

Animal models have been used to carry out cellular senescence‐targeted senotherapeutic medications, and they have shown better and functional status.^95^, ^96^ Senescent cell antiapoptotic pathways or SCAPs are inhibited by senolytics, which kill senescent cells in a targeted manner. Dasatinib with quercetin (D & Q), when combined synergistically, were the first medicines regarded as senolytics discovered in 2015 under the direction of Zhu et al.^97^ D is originally a chemotherapeutic medication for the management of chronic myeloid leukemia that shows resistance to imatinib, another tyrosine kinase inhibitor. It inhibits numerous tyrosine kinases with broad targeting of Src kinases in its action. Q on the other hand, is a nonsynthetic and nonspecific kinase inhibitor that works on PI3K/AKT pathways, in addition to BCL‐2, insulin/IGF‐1, and HIF‐1 SCAPs components. It also shows a senolytic action, presumably as a result of the inhibition of many SCAP genes (like PI3K and other kinases), and it targets a number of SCAPs pathways.^97^, ^98^, ^99^ When taken together, D + Q are complementary and result in greater senolysis. They also reduce the burden of senescent cells and human tissue SASP after 2 days of administration.^97^, ^100^ To make it easier to organize larger efficacy trials, an affirmative randomized, placebo‐controlled study of D + Q in IPF patients was conducted to assess the efficacy and ability to tolerate D + Q compared to placebo. The prescribed drug dosage schedule (108/108 doses) and the intended assessments (60/60) were completed by all participants. No significant side effects connected to D + Q were addressed, despite the fact that the placebo contained fewer total mild AEs. The majority of AEs with D + Q treatment are typical in patients with IPF or expected D AEs. Anxiety and sleep problems were overrepresented in the D + Q treatment. Before and after intermittent D + Q, fragility, pulmonary function, and physical function were examined; although underpowered to detect differences, these variables do not seem to be significantly different across groups. It is possible and typically well tolerated to provide D + Q intermittently to people with IPF. To establish the safety and effectiveness of D + Q in IPF patients, additional research, like a bigger RCT, is required.

## 4 SYMPTOMATIC TREATMENT OF IPF

There aren't many well‐powered trials that have been done, hence managing an IPF exacerbation is not yet reported. Most respiratory doctors test pulsing of methylprednisolone (0.5–1 g a day over 3 days), while anecdotal reports have also mentioned the use of cyclophosphamide and rituximab.^101^ Although often used, intravenous steroids have not been the subject of a randomized, placebo‐controlled trial. The responsiveness to corticosteroids based on accumulating dose in IPF acute phases was examined in a retrospective trial. Higher doses of steroids were favorable in ILDs except in IPF, according to the authors.^102^ However, in a different investigation, steroid treatment in the acute phase of IPF was linked to greater death rate.^103^ One more retrospective study demonstrated that early reduction of steroids after pulses provided superior results.^104^ Corticosteroid therapy had a negative influence on IPF patients, especially on diabetes and osteoporosis,^105^ as well as in randomized, double‐blind, placebo‐controlled trials that have clarified that adding prednisone to azathioprine and N‐acetylcysteine (NAC) increases mortality rate,^106^ so corticosteroids aren't recommended to patients with IPF, as it is known that the corticosteroids have anti‐inflammatory effect and recent studies have shown IPF's secondary role of inflammation in addition to the primary role of fibrosis in the pathogenesis of IPF,^106^ so we recommended in using antifibrotic agents instead of anti‐inflammatory agents in IPF. Regardless of the paucity of high‐quality proofs, the most recent updates of IPF management guidelines continue to recommend using steroids in the treatment of acute phases.^107^ Prospective randomized trials are therefore required. Other immunosuppressive medications are frequently used in some nations.^106^

## 5 THE ROLE OF SOTATERCEPT IN THE TREATMENT OF PH IN IPF

There is an urgent need for better treatments for PH in the context of IPF since patients with advanced ILDs who also have coexisting pulmonary vascular disease have worse outcomes than they would have with either diagnosis alone.^107^

**Figure 2:**
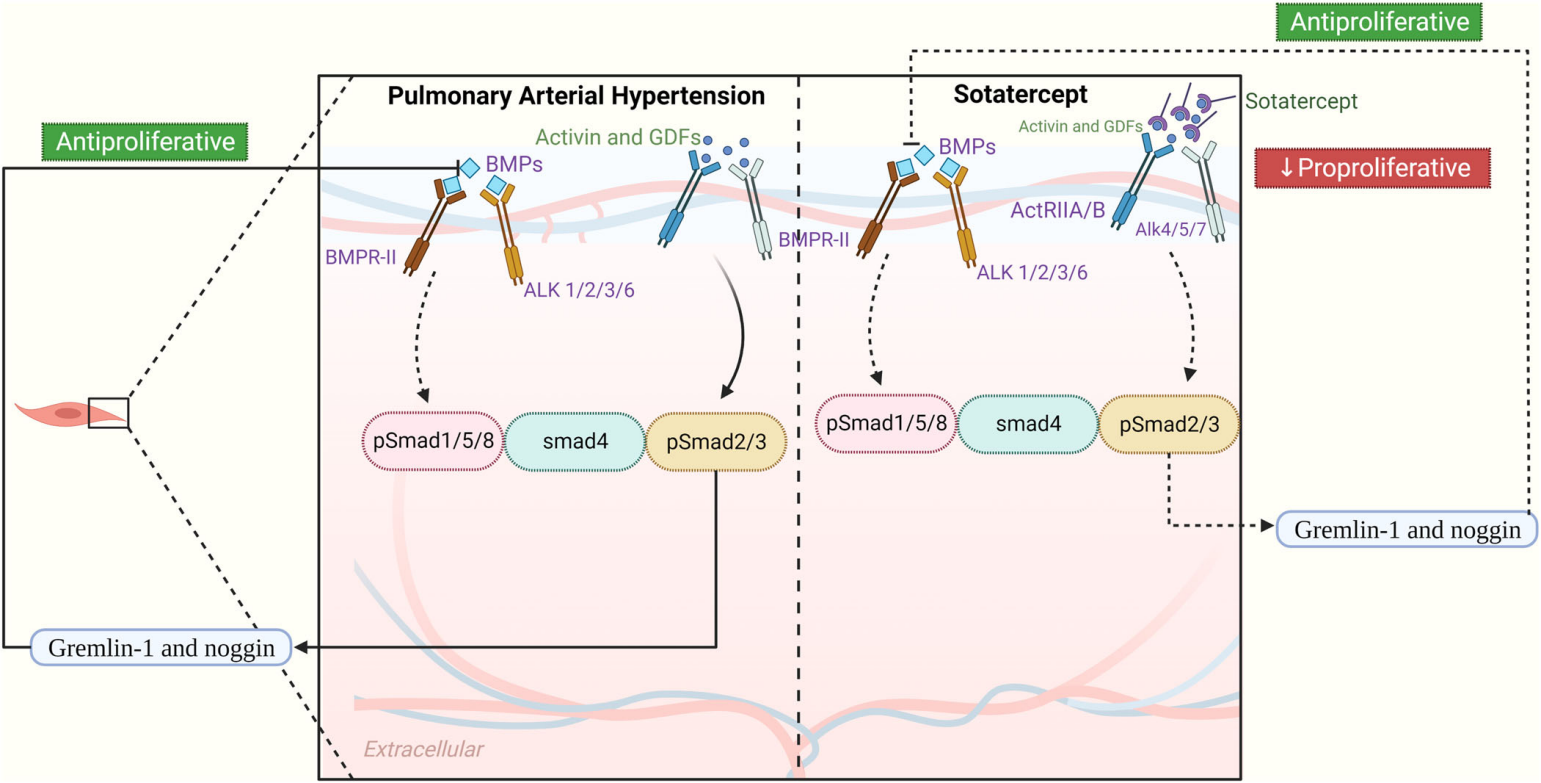
Proposed mechanism of action for Sotatercept in pulmonary arterial hypertension. The proposed mechanism of action for Sotatercept involves rebalancing growth‐promoting and growth‐inhibiting signaling in pulmonary arterial hypertension. In pulmonary vascular smooth muscle and endothelial cells, there is dysregulation of the BMP receptor type II (BMPR‐II)‐Smad1/5/8 signaling pathway, which leads to an imbalance between proliferative and antiproliferative signaling pathways. Downregulation of the BMPR‐II–Smad1/5/8 pathway leads to increased production of activin ligands (e.g., activin A, GDF8, and GDF11), which in turn up‐regulate the ActRIIA–Smad2/3 pathway. This pathway activation, indicated by increased phosphorylated Smad (pSmad)2/3 activity, promotes the expression of endogenous BMP antagonists, gremlin‐1 and noggin. Gremlin‐1 and noggin subsequently reduce BMP–Smad1/5/8 signaling, resulting in a decrease in antiproliferative signaling. This shift favors proproliferative activin–Smad2/3 signaling, leading to pulmonary vascular remodeling.

A newly developed fusion‐protein, named sotatercept, attempts to balance pro‐ and antiproliferative (BMPR‐II‐ and ActRIIA‐mediated) signals by binding to and sequestering a subset of TGF superfamily ligands (Figure 2). Sotatercept has been demonstrated to stop right ventricular remodeling and pulmonary arterial wall remodeling in PH preclinical models.^108^ In a phase 2 trial (NCT03496207), sotatercept significantly decreased vascular resistance in the lungs of patients with PH receiving background therapy. Currently, sotatercept is the subject of ongoing clinical studies (NCT03738150, NCT04576988, NCT04811092, NCT04896008).

A significant clinical research program involving patients with PH, including the phase 2 PULSAR project, is evaluating the clinical effectiveness and safety of sotatercept when it's added to PH medication.^109^, ^110^ The sotatercept medication significantly decreased pulmonary vascular resistance (PVR) during the 24‐week placebo‐controlled treatment phase of the PULSAR study from baseline when compared to placebo. In addition, in comparison with placebo, sotatercept increased the levels of 6MWD and the N‐terminal pro‐B‐type natriuretic peptide (NT‐proBNP).

In a recent multicenter, double‐blind, phase 3 trial involving adults with pulmonary arterial hypertension, Sotatercept enhanced exercise capacity more than placebo in pulmonary arterial hypertension patients who were receiving stable background therapy, as assessed by the 6‐min walk test.^111^

An ongoing innovative exploratory investigation called the SPECTRA project (NCT03738150) aims to assess the influence of sotatercept through invasive cardiopulmonary exercise testing (iCPET). Hemodynamics, exercise tolerance, and capacity showed encouraging outcomes in this preliminary examination of participants in the continuing SPECTRA trial. Safety was comparable with other reports in patient populations with PH and other conditions. These outcomes emphasize the sotatercept's clinical effectiveness and potential as a brand‐new therapy for PH patients.^112^

## 6 DISCUSSION

Undoubtedly, significant strides have been achieved in the field of pharmacological treatments for IPF over the past decade. This progress has been highlighted by the introduction of two crucial antifibrotic agents, namely pirfenidone and nintedanib, representing a momentous breakthrough in the ongoing battle against this condition. These advancements owe their success to an enhanced comprehension of the disease's pathophysiology. Once considered rare and “idiopathic,” the global rise in IPF incidence can be attributed to refined diagnostic criteria and increased clinical awareness. The recent accumulation of knowledge concerning genetic and nongenetic risk factors associated with this diverse disease has paved the way for further research endeavors. These efforts involve the development of new medications or the repurposing of existing ones to address the challenges posed by this debilitating condition.

We think the evolving understanding of IPF is significant. This progressive lung disease, characterized by fibrotic alveolar remodeling and irreversible loss of pulmonary function, has spurred a shift in focus from symptomatic management to the development of effective treatments. Current research has brought antifibrotic agents like nintedanib and pirfenidone to the forefront of IPF management. These drugs offer hope for patients by slowing disease progression and improving lung function. And our study aligns with existing literature regarding the efficacy and safety profiles of Pirfenidone and Nintedanib in treating IPF.

Regarding Pirfenidone, several studies, including ASCEND^113^ and CAPACITY,^114^ have assessed its effectiveness and safety over extended periods. In comparison to other research articles, the findings align with our study in terms of having the PFD's ability to slow down disease progression and improve lung function in IPF patients.^115^

Nintedanib, with its broad mechanism of action targeting tyrosine kinase receptors, has also shown promise in slowing the decline of lung function in IPF patients. The FDA approval of NDB for IPF in the Europe and United States based on trials like INPULSIS and INSTAGE highlights its clinical success.^58^, ^59^, ^60^, ^61^ In comparison to other studies, the positive outcomes seen in these trials are in line with the findings of the newest ones.^116^ Additionally, emerging candidates such as Saracatinib, pamrevlumab, and rhPTX‐2 are under investigation, showcasing the dynamic nature of IPF research. The investigational therapies, including BI 1015550, ziritaxestat, PBI‐4050, PLN 74809, BMS‐986020, and TD139, are intriguing prospects. However, their potential benefits must be carefully weighed against safety considerations. For example, regarding PBI‐4050, In comparing our review with another clinical trial that have been conducted on IPF patients they were treated with 800 mg of PBI‐4050 daily, with or without nintedanib or pirfenidone, for the same period which is a 12‐week period, both studies shared the common objective of assessing the safety, tolerability, and efficacy of PBI‐4050, either as monotherapy or in combination with existing IPF treatments (nintedanib or pirfenidone), within a 12‐week duration and among IPF patients. Also, a strong safety profile for PBI‐4050 when administered alone or with Nintedanib is reported. Additionally, it's found that there lie promising outcomes for PBI‐4050, particularly when used in conjunction with nintedanib. Furthermore, further investigation of PBI‐4050 alone and in combination with nintedanib is needed for continued research in this area to better understand its safety and efficacy in IPF management, particularly in different combination therapies.^117^, ^118^

The concept of senolytic therapy, combining D and Q, represents an innovative approach. However, it is important to acknowledge the need for extensive clinical trials to validate its effectiveness and safety in IPF. Additionally, the positive outcomes observed with Sotatercept in preclinical models and clinical trials for treating PH in IPF patients hold promise, but further research is imperative to comprehensively assess its efficacy and safety. The complexities of managing IPF exacerbations highlight the need for evidence‐based guidelines. High steroid doses and pulse methylprednisolone have been common approaches, but their efficacy remains uncertain. Emerging evidence suggesting early steroid reduction as an alternative approach underscores the ongoing debate within the research community.

In the context of IPF and coexisting PH, the need for effective treatments is pressing. Patients with both conditions tend to face worse outcomes than those with either alone. A promising candidate, sotatercept, a fusion protein, aims to rebalance signaling pathways involved in PH. Preclinical models show it can halt disease progression. Clinical trials reveal sotatercept's potential, reducing lung vascular resistance and improving patient parameters like the 6‐min walk distance and NT‐proBNP levels. An ongoing study, SPECTRA, reinforces these findings. In comparison with another clinical trial study, The STELLAR trial is a double‐blinded, randomized phase 3 trial looking at the safety and efficacy of sotatercept on top of background therapy for PH. it is evident that both underscore the promising therapeutic potential of sotatercept for PH, illustrating its efficacy and clinical improvement in patients. These studies, through their robust methodological design, including double‐blind and placebo‐controlled methods, emphasize the reliability and validity of the clinical findings. Additionally, both studies incorporate patients who are already on background or standard therapy for PH, providing a consistent baseline for evaluating sotatercept's effectiveness. A recurring theme in both studies is the emphasis on the clinical improvement and efficacy of sotatercept, demonstrated by its ability to increase exercise capacity and decrease PVR. Furthermore, safety is a pivotal focus in both studies, where the occurrence of adverse effects is meticulously noted and compared to those in placebo groups or other existing conditions, adding another layer of depth to the comprehensive evaluation of sotatercept's therapeutic potential. The unified understanding from these studies renders a holistic view, elucidating sotatercept's role, and potential in the medical field, thus forming a collective discourse on its viability as a novel treatment for PH.^119^ While promising, the research is based on small sample sizes, necessitating longer‐term assessments, and larger trials to establish sotatercept as a standard PH treatment in IPF. Nonetheless, these results offer hope for improved care in this challenging dual diagnosis.

## 7 CONCLUSION

Recent clinical trials have focused on developing effective treatments including antifibrotic agents (nintedanib and pirfenidone) and promising options like Saracatinib, pamrevlumab, and rhPTX‐2. Investigational therapies such as BI 1015550, ziritaxestat, PBI‐4050, PLN 74809, BMS‐986020, and TD139 require further evaluation. The combination of D and Q shows potential as a senolytic therapy. The SPECTRA trial supports its effectiveness, but more studies are required for the evaluation of its efficacy and safety. In this review, we provided an updated review of the current clinical trials and future treatment modalities regarding IPF. To find effective treatment for IPF, the future studies should provide in‐depth understandings of the physiological and pathogenic mechanisms with a focus on identifying biomarkers to evaluate the long‐term effectiveness of drugs.

## AUTHOR CONTRIBUTIONS

**Dalia D. Hadi**: Conceptualization; resources; visualization; writing—original draft. **Mohammed Dheyaa Marsool Marsool**: Conceptualization; visualization; writing—original draft; writing—review and editing. **Ali Dheyaa Marsool Marsool**: Visualization; writing—original draft; writing—review and editing. **Neel Vora**: Visualization; writing—original draft; writing—review and editing. **Sajjad G. Al‐Badri**: Writing—original draft; writing—review and editing. **Nabeel H. K. Al‐Fatlawi**: Writing—original draft; writing—review and editing. **Ameer F. A. Al Wssawi**: Writing—original draft; writing—review and editing. **Abdullah M. T. Al‐Ibraheem**: Writing—original draft; writing—review and editing. **Khadija A. Hamza**: Writing—original draft; writing—review and editing. **Priyadarshi Prajjwal**: Supervision; writing—original draft; writing—review and editing. **Mohammed A. Mateen**: Writing—review and editing. **Omniat Amir**: Writing—review and editing.

## CONFLICT OF INTEREST STATEMENT

The authors declare no conflict of interest.

## ETHICS STATEMENT

The authors have nothing to report.

## Data Availability

The authors confirm that the data supporting the findings of this study are available within the article.
